# Multidimensional Transport Experiment and Simulation of Chloride Ions in Concrete Subject to Simulated Dry and Wet Cycles in a Marine Environment

**DOI:** 10.3390/ma16227185

**Published:** 2023-11-16

**Authors:** Hao Xu, Zixi He, Jianxin Li, Shuangxi Zhou

**Affiliations:** 1 School of Civil and Engineering Management, Guangzhou Maritime University, Guangzhou 510725, China; jianxinl@126.com; 2 Guangdong Provincial Key Laboratory of Green Construction and Intelligent Operation & Maintenance for Offshore Infrastructure, Guangzhou Maritime University, Guangzhou 510725, China; 3 School of Civil Engineering and Architecture, East China Jiaotong University, Nanchang 330013, China; zxhe1995@hotmail.com

**Keywords:** concrete, dry and wet cycles, chloride ion content, microstructure, multidimensional transport model

## Abstract

Chloride ion erosion is an important factor affecting the durability of marine engineering concrete. In particular, concrete structures in wave splash and tidal zones are subjected to dry and wet cycles and multidimensional diffusion of chloride ions. To investigate the intricate diffusion of chloride ions within concrete under these dynamic conditions, we devised a comprehensive experiment. This experiment encompasses multiple dimensions, involving dry and wet cycles, as well as static immersion. The experiment intends to reveal how chloride ions are distributed in the concrete and clarify the changes that occur in its microstructure. Based on Fick’s second law, the multidimensional diffusion model of chloride ions in concrete under the dry and wet cycles and static immersion was established by comprehensively considering the effects of chloride ion exposure time, environment temperature, relative humidity, and the action of dry and wet cycles. The results show that, under the same conditions, the chloride content in concrete decreases with the increase in penetration depth but increases with the increase in the chloride diffusion dimension and exposure time. Dry and wet cycles and multidimensional diffusion of chloride ions increase the development of cracks and pores in the concrete structure and generate large quantities of C_3_A·CaCl_2_·10H_2_O, which will exacerbate the chloride ion transport rate and penetration depth of concrete. Under the same exposure time and penetration depth, the chloride ion content in concrete under two-dimensional (2D) and three-dimensional (3D) diffusion under dry and wet cycles was 1.09~4.08 times higher than that under one-dimensional (1D) diffusion. The correlation coefficients between the simulation results of the multidimensional transport model of chloride ions in concrete under multi-factor coupling and the experimental results were all greater than 0.95, and the model can be utilized to predict the distribution of chloride ion concentration in concrete.

## 1. Introduction

With the implementation of China’s marine power strategy and the development of the marine economy, the demand for diverse types of marine engineering concrete structures is rising. Numerous aggressive ions in the ocean, especially the presence of a large number of chloride ions, which is the main factor causing the corrosion of steel reinforcement and leading to the deterioration of concrete [1,2,3,4,5,6], it is necessary to pay attention to the effect of chloride ions on the durability of marine engineering concrete. Marine engineering concrete structures can be classified based on their contact with seawater into four zones: atmospheric exposure, wave splash, tidal, and submerged [7]; the degree of chloride ion erosion varies among these regions. Concrete structures in the wave-splash zone and tidal zone are in alternating wet and dry environments, and microcracks within the concrete are continuously generated and enlarged by the dry and wet cycles, which accelerates the erosion of chloride ions and leads to a reduction in the durability of the concrete [8,9,10,11]; the chloride ion erosion in the concrete under the action of dry and wet cycles has always been a focus of the research of scholars at home and abroad, and a lot of research results have been achieved in the experiment and numerical simulation of chloride ion transport in concrete under the action of dry and wet cycles.

In the experimental study of chloride ion transport in concrete under the action of dry and wet cycling, Neville [1] investigated the process and mechanism of chloride ion transport in concrete under dry and wet cycles. Zhang et al. [12] summarized the research progress of marine concrete under the combined action of chloride erosion and dry–wet cycles. Wei et al. [13], Ye et al. [14], and Backus et al. [15] investigated the chloride ion migration process and its corrosion process on steel reinforcement under the alternating state of dry and wet cycles in concrete and verified that dry and wet cycles would accelerate the migration of chloride ions. Wu et al. [16] investigated the one-dimensional erosion characteristics of chloride ions in concrete under the action of dry and wet cycles by using a 3% NaCl solution for 8 h wetting and 8 h drying of concrete at room temperature. They found that the chloride content in the dry and wet cycle condition tends to be stabilized at a distance of 10–15 mm from the surface of the concrete and that the chloride content tends to be stabilized at a distance of 5–10 mm from the surface of the concrete in the full-immersion condition. Many scholars agree that the dry-to-wet ratio is an important factor affecting the chloride ion resistance of concrete. By designing dry and wet cycles experiments at different dry-to-wet ratios, Xu et al. [17,18] and Hong et al. [19] concluded that an increase in the dry-to-wet ratio would result in an accelerated chloride ion transport rate in concrete. The experiments of Xu et al. [17,18] demonstrated that the fastest chloride ion transfer rate of concrete occurred under dry and wet cycles with a 5:1 dry-to-wet ratio. Hong et al. [19] later found that a higher ratio of 11:1 further accelerated the transfer of chloride ions in the concrete. However, Sutrisno et al. [20], Li et al. [21], and Huang et al. [22] concluded that concrete with a smaller dry-to-wet ratio is less resistant to chloride erosion under dry and wet cycling. Wang et al. [23] concluded that the diffusion coefficient of chloride ions was highest when using a dry-to-wet ratio of 3:1 by observing the transport characteristics of chloride ions in concrete under four dry-to-wet ratios such as 1:1, 3:1, 5:1, and 7:1. Cao et al. [24] investigated the chloride ion transport process in coral concrete under dry and wet cycling and explored the corrosion pattern of steel reinforcement in coral concrete. Meanwhile, the increase in the number of wet and dry cycles will significantly accelerate the diffusion of chloride ions [25]. Lu et al. [26] studied the effect of the concrete mix ratio, dry-to-wet ratio, and exposure time on chloride ion penetration into concrete under the action of dry and wet cycles; the depth of chloride ion penetration increased with the increase in dry-and-wet-cycle air-drying time and exposure time. Xu et al. [27] and Marcos-Meson et al. [28] concluded that carbonation will accelerate the chloride ions transport in concrete under dry and wet cycles. Dong et al. [29] investigated the chloride ion transport and service life of aeolian sand concrete under dry–wet cycles. The transport of chloride ions under wet and dry cycling is inevitably affected by the pore structure of concrete. Arya et al. [30,31] analyzed the effect of factors affecting the pore structure of concrete, such as the water–cement ratio, cement type, and slag-powder admixture, on the distribution of chloride ions under wet and dry cycling. The diffusion rate of chloride ions in concrete is temperature-dependent: as the temperature increases, the diffusion rate is faster [32,33,34]. For this reason, Chen et al. [35] investigated the corrosion resistance of concrete against chloride ions under the effect of dry–wet cycling and temperature coupling; higher dry and wet ratios and variable temperature cycling will accelerate the transport of chloride ions in concrete. In recent years, due to the application of recycled aggregates, Chen et al. [36] investigated the transport pattern of chloride ions in recycled concrete under the action of dry and wet cycles. Du et al. [37] investigated the sulphate resistance of recycled aggregate concrete under sulphate wetting–drying cycles. Lu et al. [38] studied the dynamic properties and chloride resistance of basalt and polypropylene-fiber-reinforced recycled aggregate concrete based on the experiment.

In terms of modeling studies of chloride ion transport in concrete under dry and wet cycling, Ababneh et al. [39] established the diffusion and convection equations for chloride ions by dividing the chloride ion transport in concrete under dry and wet cycling into diffusion and convection components. Zhang [40] developed a diffusion convection model for chloride ions under dry and wet cycling conditions by considering the difference in water dispersion coefficients under alternating dry and wet conditions. Huang et al. [41] proposed chloride transport models for the convective diffusion zone and diffusion zone, respectively, based on the transport mechanism of chloride ions in concrete under dry and wet cycling. Guan et al. [42] modeled the one-dimensional transport of chloride ions in concrete under dry and wet cycles by taking into consideration the effects of temperature, concrete age, anthropogenic factors, and wind speed. Soive et al. [43] simulated one-dimensional transport properties of chloride ions in concrete under dry and wet cycling based on physical and chemical coupled ion–moisture transport models. Cao et al. [44] investigated the two-dimensional transport characteristics of chloride ions in concrete under dry and wet cycling and developed a two-dimensional transport model of chloride ions in concrete with full consideration of porosity, curvature, concrete saturation, and cement hydration. For the diffusion of chloride ions in cracked concrete under dry and wet cycling, Paul et al. [45] investigated the chloride ingress in cracked and uncracked strain-hardening, cement-based composite under dry and wet cycling, and the results show that the cracked condition will accelerate the migration of chloride ions within the concrete. Ye et al. [46] proposed a chloride ion transport model to characterize cracked concrete under dry and wet cycling and validated it using experiments. Lu et al. [47] investigated the penetration pattern of chloride ions in concrete under the coupled effects of cracking and dry and wet cycling and established an equivalent diffusion model for chloride ions in cracked concrete under dry and wet cycling conditions. Lai et al. [48] studied the effect of crack width on chloride ion penetration in reinforced concrete beams under dry and wet cycles and proposed the simulation model to predict the relationship between the crack width and influence coefficient.

From the above studies, it can be seen that domestic and foreign researchers have conducted a lot of studies mainly on the one-dimensional (1D) diffusion test and simulation of chloride ions in concrete under the action of dry and wet cycles; however, the concrete structures of marine engineering are often subjected to two-dimensional (2D) or three-dimensional (3D) diffusion of chloride ions. For this purpose, this paper carries out indoor modeling tests on the multidimensional transport of chloride ions in concrete under the action of dry and wet cycles and static immersion by simulating the concentration of chloride ions in seawater in the Tongan District, Xiamen City, China and tests the microstructural changes in concrete before and after the dry and wet cycling by using Scanning Electron Microscopy (SEM) and X-ray Diffractometry (XRD) to study the effects of dry and wet cycling and multidimensional diffusion on the transport pattern of chloride ions in concrete. On this basis, the multi-dimensional transport model of chloride ions under dry and wet cycles and static immersion is established based on Fick’s second law by considering the effects of dry and wet cycles, time-varying diffusion of chloride ions, ambient temperature, and relative humidity and is verified using the experimental results. The research results can provide a reference for the study of multi-dimensional chloride ion transport mechanisms and the improvement of chloride ion resistance of marine engineering concrete structures under multi-factor coupling.

## 2. Experimental Procedures

### 2.1. Raw Materials and Mixed Proportion

The cement used in this test is Longlin-brand P.O 52.5 ordinary silicate cement produced by Fujian Longlin Group Co., Ltd., Longyan city, China; the product complies with GB 175-2007 [49], and its material composition and physical properties are given in Table 1. Coarse aggregate is natural gravel produced by Taining County Wanxing Building Materials Co., Ltd., Sanming Ctiy, China with an apparent density of 2630 kg/m^3^, crushing index of 8.7%, and particle grading of 5 mm~25 mm. The fine aggregate is natural river sand produced by Xiamen Shunlei Building Material Co., Ltd., Xiamen City, China with an apparent density of 2590 kg/m^3^ and a fineness modulus of 2.5. The fly ash adopted was grade I fly ash produced by Hejin Longjiang Fly Ash Development and Utilization Co., Ltd., Hejin City, China and its performance meets the requirements of GB/T 1596-2017 [50]. S95 slag powder is produced by Leting County Changxu Building Materials Co., Ltd., Tangshan Ctiy, China and its performance meets the requirements of GB/T 18046-2017 [51]. The water is industrial tap water from Xiamen, Fujian Province, and the admixture is a polycarboxylic acid retarder to improve concrete compatibility with a water reduction rate of 18%.

The design strength grade of concrete for this experiment is C55, and the mix proportion is in reference to the construction mix proportion of a cross-sea bridge in Xiamen; the water-to-binder ratio is 0.3, and the mix proportion of concrete is shown in Table 2.

### 2.2. Specimen Preparation

This experiment mainly simulates the corrosive effect of chloride ion diffusion and diffusion dimension on concrete under the dry and wet cycles in the marine environment, and a total of 80 cubic concrete specimens of 100 mm in size, 3 rectangular concrete specimens of 100 mm × 100 mm × 400 mm, and 6 cylindrical concrete specimens of Φ100 mm × 50 mm were fabricated. And, 3 cubic concrete specimens were used to determine the compressive strength at 7 and 28 days, 3 rectangular concrete specimens to measure the flexural strength at 28 days, 3 cylindrical concrete specimens to evaluate the chloride diffusion coefficient using the Rapid Chloride Migration Coefficient (RCM) method, and 54 cubic concrete specimens to conduct diffusion of chloride ions tests.

**Figure 1:**
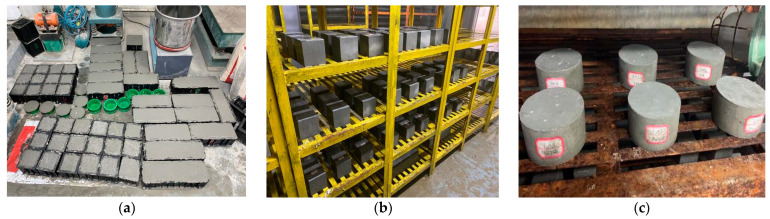
Concrete specimen molding and curing: (**a**) Concrete specimen modeling; (**b**) Cubic specimen maintenance; (**c**) RCM specimen maintenance.

To prevent interference from other factors, we used raw materials from the same batch and poured the specimens on the same day. The temperature of the curing room is (20 ± 2) °C, the relative humidity is greater than 95%, and the molding of concrete specimens and their maintenance are shown in Figure 1.

**Figure 2:**
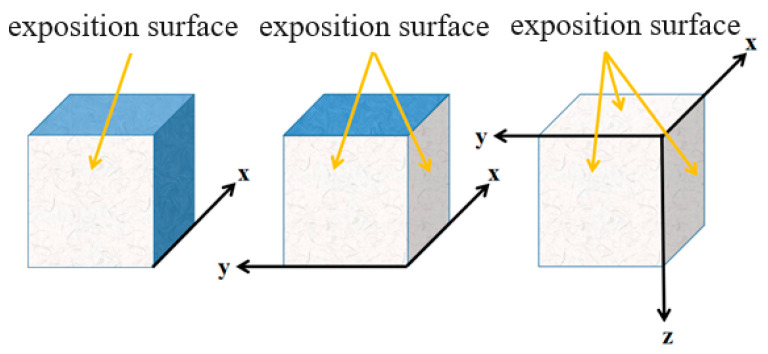
The treatment of chloride ion diffusion experiment specimens.

Before the chloride ion diffusion test, the concrete specimens should be pretreated. For the 1D diffusion of cubic concrete specimens, 5 faces are coated with epoxy resin and only one exposition surface is kept in contact with the salt solution; for the 2D diffusion of cubic concrete specimens 2, adjacent surfaces are kept as exposition surfaces; and for the 3D diffusion of cubic concrete specimens, 3 two-by-two surfaces are kept as exposition surfaces, and the rest of the surfaces are encapsulated with epoxy resin as shown in Figure 2.

### 2.3. Chloride Ion Diffusion Test

In order to make the immersed salt solution as close as possible to the real marine environment, in the experiment, the seawater sampling was carried out in the Tongan District, Xiamen City; the number of samples was three, respectively, to determine the concentration of Cl^−^ and SO_4_^2−^ in seawater and to take the average of its value as the concentration of the salt solution for the experiment. High-purity industrial chemicals were used for the configuration, and a salt solution of 2.8% NaCl + 0.29% Na_2_SO_4_ was used for the dry and wet cycle tests. Referring to the tidal changes in Xiamen in recent years, a dry and wet cycle system of 12 h natural drying + 12 h solution immersion (the dry and wet ratio is 1:1) as a cycle was selected as a comparison group, while the concrete specimens were put into the salt solution for static immersion for 30 days, 60 days, and 90 days, respectively. To ensure the stability of the ion concentration in the solution, the salinity change of the solution was recorded weekly using a seawater salinometer and the solution was replaced once a month for the treatment. The different dimensions of chloride ion diffusion conditions and numbers are shown in Table 3.

### 2.4. Experimental Method

(1) Compressive and flexural strength test

The compressive and flexural strength of concrete was measured according to GB/T 50081-2019 [52]. A CXYAW-2000E micro-control pressure testing machine is used to test the compressive strength of concrete. A WAW-100B microcomputer-controlled, electro-hydraulic servo universal testing machine is used to evaluate the flexural strength of concrete. Since the tested specimens are non-standard specimens, according to the literature [52], the compressive strength test value should be multiplied by the size conversion factor of 0.95 for the non-standard specimens of 100 mm in size. The flexural strength test value should be multiplied by the size conversion factor of 0.85 for the non-standard specimens of 100 mm × 100 mm × 400 mm. The compressive strength and flexural strength of concrete at 28 days are shown in Table 4.

(2) Chloride penetration resistance test

The migration coefficient of chloride ions in concrete was measured according to the requirements of GB/T 50082-2019 [53], and the migration coefficient of chloride ions was measured by using a RCM-10 concrete chloride ion diffusion-coefficient tester and the grade of the anti-chlorine ion penetration performance of concrete was classified according to JGJ/T 193-2009 [54]. The chloride ion diffusion coefficients of 28 d and 84 d of concrete designed for this test were measured by RCM to be 4.2 × 10^−12^ m^2^/s and 1.7 × 10^−12^ m^2^/s, respectively, and the grade of chloride ion penetration resistance of concrete was RCM-IV, which means the concrete has good chloride penetration resistance.

(3) Chloride ion content test

**Figure 3:**
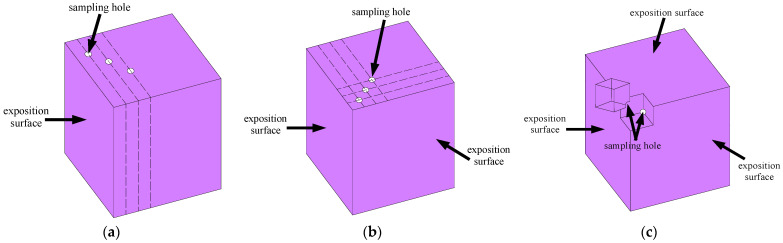
Concrete specimen sampling schematic: (**a**) 1D exposition; (**b**) 2D exposition; (**c**) 3D exposition.

The water-soluble chloride content of the concrete was measured after the concrete specimens had been subjected to diffusion of chloride ions for 30, 60, and 90 days. Concrete powder was collected at 1 mm, 10 mm, 20 mm, and 30 mm from the diffusion surface, respectively, and the sampling locations of concrete under 1D, 2D, and 3D diffusion of chloride ions are shown schematically in Figure 3. Among them, 1D and 2D samples were collected using hand-held impact drills, and when the impact drills were used to collect concrete powder, in order to prevent the edges of the concrete specimens from breaking, a certain amount of pressure was applied to the other four neighboring surfaces during the sampling process; 3D-exposed material was sampled using a marble cutter for cutting and grinding. As for 3D-exposition material sampling, the position to be sampled was first marked on the concrete surface, and then the cubic nuggets as shown in Figure 3c were cut out from the concrete specimens; the inner corners of the cubic nuggets were ground with a grinder. The powder was extracted from the concrete specimens using hand-held impact drills at the position corresponding to the inner corner of the cubic nuggets, and the powder obtained in both ways was collected together. The collected powder was ground until it could pass through a sieve with a nominal diameter of 0.16 mm, baked in an oven at 105 °C ± 5 °C for 2 h, and then placed in a desiccator to cool to room temperature. The water-soluble chloride ion content of concrete under different dimensions of diffusion was measured according to JGJ/T322-2013 [55]. Three specimens were tested for each condition; if the dispersion of the experiment results was large, the number of specimens was increased to ensure the accuracy of the experiment results. The average of the experiment results of the three specimens was used for analysis.

(4) Microstructure observations

A ZEISS Sigma 300 scanning electron microscope (SEM) from Germany and Rigaku Ultima IV X-ray diffractometer (XRD) from Japan were used to observe the microstructure and chemical composition of the concrete, respectively, and the sampling point was 5 mm from the concrete surface.

When SEM was used to observe the microstructure of concrete, the samples were recovered from fractured surfaces to avoid destroying the internal material morphology of the samples. Due to the poor conductivity of the concrete, which will affect the clarity of the observation, it is also necessary to use the Quorum SC7620 sputtering coater to spray gold on the sample. The sample was fixed on the conductive tape when observing the microstructure of the concrete.

When XRD was used, a concrete block with a thickness of not less than 0.1 cm was glued to a glass sample stage, a Cu anticathode with a wavelength of 0.154056 nm was used, the test angle was from 5° to 90°, and the scanning speed was selected to be 2°/min.

## 3. Experiment Results and Discussion

### 3.1. Chloride Ion Concentration Distribution

**Figure 4:**
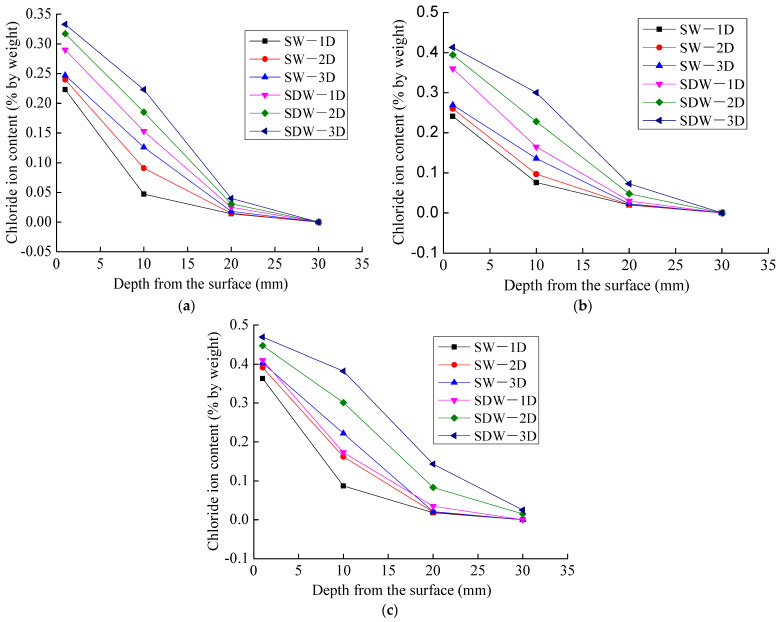
Distribution of chloride ion concentration in concrete under different conditions: (**a**) 30 days; (**b**) 60 days; (**c**) 90 days.

The distribution of chloride ion concentration in concrete specimens under different exposures modes for chloride exposure times of 30, 60, and 90 days is shown in Figure 4.

As shown in Figure 4, at the same exposure time, the chloride ion content in concrete decreases with the increase in penetration depth, and the difference in chloride ion concentration decreases. Taking the 1D diffusion of chloride ions as an example, as shown in Figure 4a, when the exposure time is 30 days, the chloride ion content of the concrete at the penetration depth of 1 mm under static immersion and dry and wet cycles is between 0.223% and 0.29%, the chloride ion content of the concrete at the penetration depth of 10 mm is between 0.047% and 0.153%, and the chloride ion content of the concrete at the penetration depth of 20 mm is between 0.014% and 0.025%, respectively. The chloride content decreased by between 0.176% and 0.137% when the penetration depth increased from 1 mm to 10 mm and decreased by between 0.033% and 0.128% when the penetration depth increased from 10 mm to 20 mm.

For the same exposure time and penetration depth, the chloride ion content of concrete under dry and wet cycles was higher than that of concrete under static immersion. This is due to the fact that under dry and wet cycles, chloride ions are transported via capillary absorption and diffusion. During the immersion phase, the capillary action and the concentration difference contribute to the ingress of chloride ions into the interior of the concrete, and during the drying phase, the internal moisture of the concrete migrates outward, the chloride concentration in the pore solution increases, and the difference in the concentration gradient contributes to the migration of chloride ions towards the interior of the concrete, which leads to a higher chloride ion content in the interior of the concrete than that of the static immersion. Taking the 1D diffusion of chloride ions as an example, as shown in Figure 4b, when the exposure time of chloride ions is 60 days the chloride ion content at penetration depths of 1 mm, 10 mm, 20 mm, and 30 mm under static immersion is 0.241%, 0.076%, 0.02%, and 0%, and that of 1 mm, 10 mm, 20 mm, and 30 mm under dry and wet cycles is 0.36%, 0.165%, 0.03%, and 0%, with an increase in chloride ion content of 0.119%, 0.089%, 0.01%, and 0%. It can be seen that, although the dry and wet cycles enhance the chloride ion transport rate in the surface layer of concrete, the enhancement in the chloride ion transport effect by dry and wet cycles is subsequently weakened with the increase in the penetration depth, which indicates that the difference in chloride ion concentration is the main factor influencing the chloride ion transport rate in the interior of concrete.

As shown in Figure 4, for the same exposure time and penetration depth, the chloride ion content in concrete increases with the increase in the chloride ion diffusion dimension. The chloride ions content in concrete of 2D diffusion under static immersion is 1.05~1.94 times that of 1D diffusion, and the chloride ions content in concrete of 3D diffusion under static immersion is 1.10~2.68 times that of 1D diffusion. The chloride ions content in concrete of 2D diffusion under dry and wet cycles is 1.09~2.57 times that of 1D diffusion, and the chloride ions content in concrete of 3D diffusion under dry and wet cycles is 1.14~4.08 times that of 1D diffusion. Taking chloride ion diffusion of 90 days as an example, as shown in Figure 4c, when the penetration depth is 10 mm, the chloride ion content of 1D diffusion in concrete under static immersion is 0.087%, and the chloride ion content of 2D and 3D diffusion in concrete is 0.162% and 0.222%, which are 1.86 times and 2.55 times than the chloride ion content under 1D diffusion in concrete. The chloride ion content of 1D diffusion in concrete under dry and wet cycles is 0.173%, and the chloride ion content of 2D and 3D diffusion in concrete is 0.301% and 0.382%, which are 1.74 times and 2.21 times that of the chloride ion content under 1D diffusion in concrete. When the penetration depth reached 30 mm, chloride ions were only detected in the concrete after 90 days of 2D and 3D chloride ion diffusion under dry and wet cycles, and the chloride ion contents was 0.015% and 0.025%. It can be seen that the multidimensional diffusion of chloride ions will exacerbate the chloride transport rate in concrete and the penetration depth, so the multidimensional diffusion of chloride ions in concrete structures in marine environments cannot be ignored.

**Figure 5:**
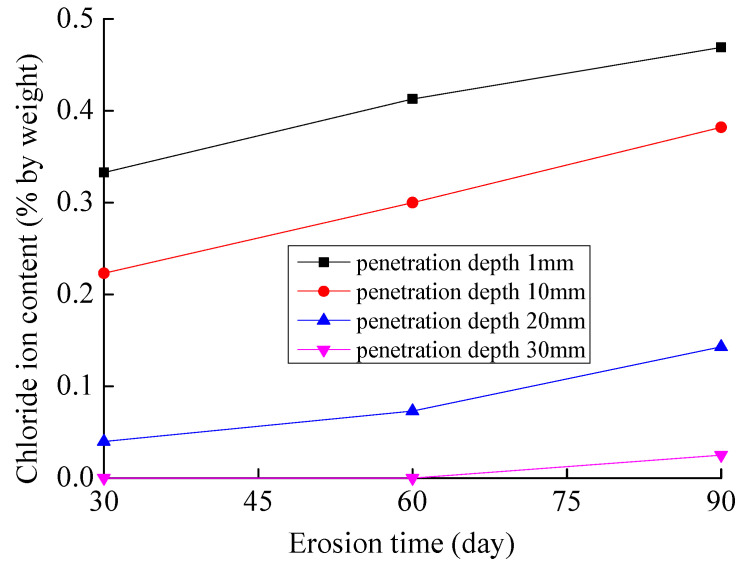
Variation of chloride content with exposure time.

For the effect of exposure time on the chloride ion content, taking the 3D diffusion of chloride ions in concrete under the dry and wet cycles as an example, the variation in chloride ion content in concrete with exposure time at different penetration depths is shown in Figure 5.

As shown in Figure 5, at the same penetration depth, the chloride ion content in concrete increases with the increase in exposure time. For the 3D diffusion of chloride ions in concrete under dry and wet cycles, when the penetration depth is 10 mm, the chloride ion content is 0.223% under dry and wet cycles of 30 days; the chloride ion content is 0.3% and 0.382% under dry and wet cycles of 60 days and 90 days, which increased by 34.5% and 71.30%, respectively. This is due to the fact that chloride ion transport in concrete is predominantly diffusive. The chloride ion concentration in concrete increases with time.

### 3.2. Change of Microstructure

**Figure 6:**
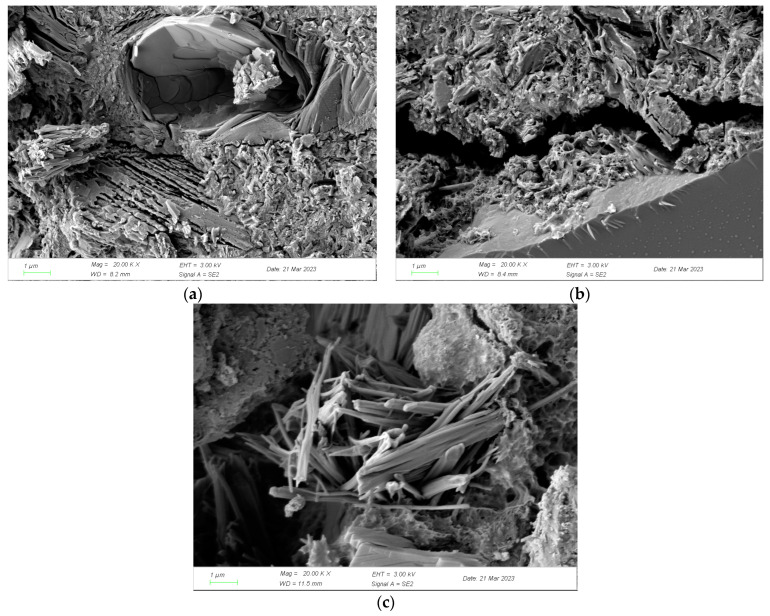
SEM results of concrete specimens in different conditions: (**a**) Comparison group; (**b**) Dry and wet cycles; (**c**) Static immersion.

The concrete without diffusion of chloride ions was used as a comparison group, and the results of electron microscope scans of concrete specimens under different working conditions and concrete without diffusion of chloride ions after 90 days of 3D diffusion of chloride ions are shown in Figure 6.

From Figure 6a, it can be seen that the concrete structure that has not been diffusion by chloride ions has good integrity and the aggregate is tightly connected to the mortar. Figure 6b illustrates that after the action of dry and wet cycles, there were obvious cracks inside the concrete structure, the pore size increased, and the connectivity was enhanced along with it, accompanied by the generation of C_3_A·CaCl_2_·10H_2_O; at the same time, the surface of the concrete also produced some fine loose particles, the boundary of the transition zone between the aggregate and the mortar was blurred, and the overall staining adhesion deteriorated, so that more chloride ions entered into the inside of the concrete, which further suggests that dry and wet cycles can significantly enhance the chloride ion transport rate in surface concrete. As shown in Figure 6c, there are many microcracks in the concrete structure, which, in turn, increases the content of chloride ions within the concrete.

**Figure 7:**
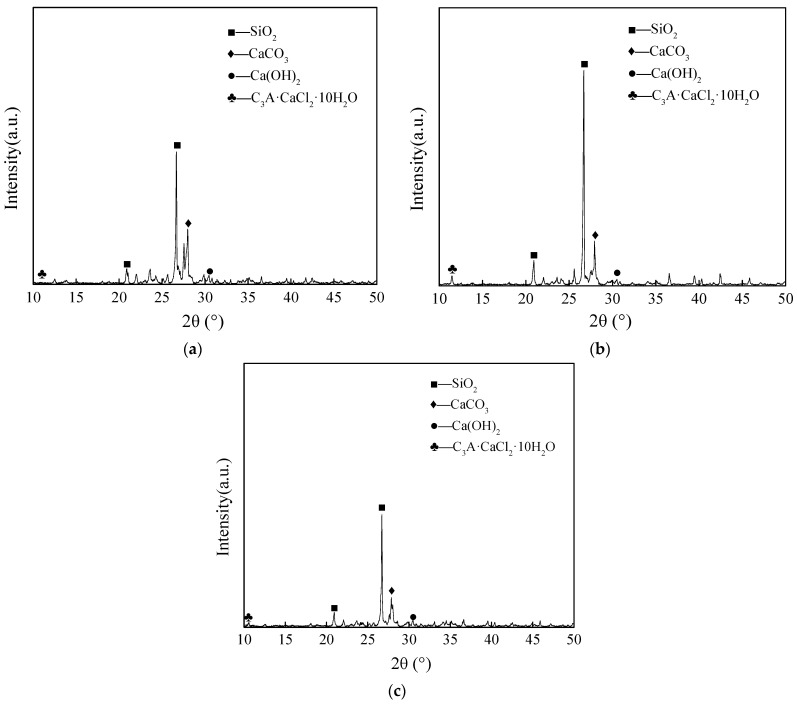
XRD results of concrete specimens under different conditions: (**a**) Comparison group; (**b**) Dry and wet cycles; (**c**) Static immersion.

The XRD physical phase change curves of concrete after 90 days of 3D chloride ion diffusion under different working conditions are shown in Figure 7. From the XRD patterns of the specimens in the dry state, it can be seen that the composition of the specimens’ physical phases mainly consisted of SiO_2_ (PDF # 85-1053), CaCO_3_ (PDF # 87-1863), Ca(OH)_2_ (PDF # 72-0156), and the C_3_A·CaCl_2_·10H_2_O (PDF # 78-1219).

After 90 days of exposure to varying conditions, we detected characteristic C_3_A·CaCl_2_·10H_2_O peaks within the concrete specimens. Notably, the C_3_A·CaCl_2_·10H_2_O peaks were more pronounced in the SDW group. This increase in peak height can be attributed to the greater number of wet and dry cycles, which accelerated damage within the concrete. The process led to pore expansion, microcracks, and differences in concentration that facilitated the transport of more chloride ions into the interior of the concrete. Consequently, chloride ions diffused more rapidly inside the concrete due to the influence of these dry–wet cycles and concentration variations, resulting in increased C_3_A·CaCl_2_·10H_2_O formation when reacting with C_3_A.

### 3.3. Numerical Simulation

The transport of chloride ions in concrete under static solution immersion is dominated by migration and satisfies Fick’s second law [56]. The 1D diffusion equation for chloride ions is shown in Equation (1).

(1) $$C(x,t)=C_{0}+(C_{s}-C_{0})\left[1-erf(\frac{x}{2\sqrt{Dt}})\right]$$

where *C*_0_ is the chloride ion concentration in the internal of concrete (unit: %), *C_s_* is the chloride ion concentration on the concrete surface (unit: %), *D* is the chloride diffusion coefficient (unit: m^2^/s), *t* is the time of concrete structures’ exposure to the environment (unit: s), *x* is the depth from the *x*-axis direction of the concrete surface (unit: mm), and *erf*(*u*) is the gaussian error function, which is shown in Equation (2).

(2) $$erf(u)=\frac{2}{\sqrt{\pi }}\int_{0}^{u}e^{-t^{2}}dt$$

The chloride ion diffusion coefficient is affected by concrete raw material components, service time, external environment, and other factors. A multidimensional diffusion model of chloride ions was established under multi-factor coupling conditions by considering the effects of time factor, chloride ion binding capacity, temperature, relative humidity, and concrete deterioration caused by dry and wet cycling. The diffusion coefficient of chloride ions in the model is expressed by the integrated diffusion coefficient of chloride ions.

The chloride diffusion coefficient *D*_0_ [57] for concrete at the temperature of 20 °C for 28*d* is given in Equation (3).

*D*_0_ = 10^−12.06+2.4*W*/*B*^ (3)

where *W*/*B* is the water-to-binder ratio.

For the effect of dry and wet cycles, since dry and wet cycles will lead to the deterioration of concrete materials, the deterioration effect factor *K* can be introduced when describing the chloride ion penetration of concrete materials under the action of dry and wet cycles [58], and the effective diffusion time of dry and wet cycle erosion specimens is shown in Equation (4).

*t* = *Ki* (*t_st_* + *t_gt_*) (4)

where *t* is the effective diffusion time (unit: s). *K* is the deterioration effect factor [58], because the deterioration effect factor of concrete under dry and wet cycles is a range, considering that the test time of this paper is relatively short; at the same time, according to a large number of trial calculations, the deterioration effect factor *K* in this paper is taken as 2.5. *i* is the number of dry and wet cycles, *t_st_* is the wet time of one dry and wet cycle (unit: s), and *t_gt_* is the dry time of one dry and wet cycle (unit: s).

For the effect of chloride diffusion varied with time, Thomas et al. [59] suggested that the time-influence factor *f_t_* of the chloride diffusion coefficient within concrete at the standard curing age can be expressed as Equation (5).

(5) $$f_{t}=(\frac{t_{0}}{t}{)}^{m}$$

where *t*_0_ is the 28*d* curing time (unit: s), *t* is the diffusion time (unit: s), and *m* is the time decay coefficient, which is given in Equation (6) [57].

(6) $$m=0.2+0.4(\frac{\omega_{FA}}{50}+\frac{\omega_{SG}}{70})$$

where *ω_FA_* is the percentage of fly ash to the mass of cementitious material, *ω_FA_*/50 ≤ 1, which means that the percentage of fly ash to the mass of cementitious material should be less than 50%. *ω_SG_* is the percentage of slag to the mass of cementitious material, *ω_SG_*/70 ≤ 1, which means that the percentage of slag to the mass of cementitious material should be less than 70%.

For the effect of chloride binding capacity, Perez et al. [60] proposed a linear model for chloride binding, and the chloride binding capacity influence factor *f_B_* can be expressed as Equation (7).

(7) $$f_{B}=\frac{1}{1+\frac{1}{\omega_{e}}\cdot \frac{\partial C_{B}}{\partial C_{F}}}=\frac{1}{1+\frac{\alpha }{\omega_{e}}}$$

where *ω_e_* is the volumetric ratio of evaporable water in concrete, *C_B_* is the concentration of bound chloride ions (unit: mol/L), *C_F_* is the concentration of free chloride ions in the concrete pore solution (unit: mol/L), and *α* is the slope of the line.

For the effect of environment temperature, Boddy et al. [61] proposed a formula for calculating the diffusion coefficient of chloride ions considering the environment temperature, where the temperature influence factor *f_T_* is shown in Equation (8).

(8) $$f_{T}=\exp\left[\frac{U}{R}\left(\frac{1}{T_{0}}-\frac{1}{T}\right)\right]$$

where *T* is the environment temperature; *T*_0_ is the reference temperature at age 28*d*, which is 293.15 K [62]; *R* is the gas constant, which is 8.314 J/(mol·K); and *U* is the activation energy during diffusion, which is 35 kJ/mol when the water-to-binder ratio is 0.3 according to the reference [57].

For the effect of the environment relative humidity, Bazant et al. [63] proposed a formula for calculating the diffusion coefficient of chloride ions taking into account the relative humidity, where the relative humidity influence factor *f_H_* can be expressed as Equation (9).

(9) $$f_{H}={\left[1+{\left(\frac{1-RH}{1-RH_{c}}\right)}^{4}\right]}^{-1}$$

where *RH* is the relative humidity at the time of the experiment and *RH_c_* is the critical relative humidity, generally 75%.

Taking into account the effects of the diffusion of chloride ions varied with time, the binding effect of chloride ions with concrete, the environment temperature, relative humidity, and the effect of dry and wet cycles, the chloride diffusion coefficient *D* is modified; assuming that the corrected diffusion coefficient of chloride ions is *D_f_*, the modified 1D, 2D, and 3D diffusion equations for chloride ions in the concrete are given in Equations (10)–(12) [64].

(10) $$C(x,t)=C_{0}+(C_{s}-C_{0})\cdot \left[1-erf\left[\sqrt{\frac{(1-m)x^{2}}{4t_{0}{}^{m}t^{1-m}D_{f}}}\right]\right],$$

(11) $$C(x,y,t)=C_{0}+(C_{s}-C_{0})\cdot \left[1-erf\left[\sqrt{\frac{(1-m)x^{2}}{4t_{0}{}^{m}t^{1-m}D_{f}}}\right]\cdot erf\left[\sqrt{\frac{(1-m)y^{2}}{4t_{0}{}^{m}t^{1-m}D_{f}}}\right]\right],$$

(12) $$C(x,y,z,t)=C_{0}+(C_{s}-C_{0})\left[1-erf\left[\sqrt{\frac{(1-m)x^{2}}{4t_{0}{}^{m}t^{1-m}D_{f}}}\right]\cdot erf\left[\sqrt{\frac{(1-m)y^{2}}{4t_{0}{}^{m}t^{1-m}D_{f}}}\right]\cdot erf\left[\sqrt{\frac{(1-m)z^{2}}{4t_{0}{}^{m}t^{1-m}D_{f}}}\right]\right],$$

where *D_f_* is the modified integrated diffusion coefficient for chloride ions; *m* is the time decay coefficient; and *y*, *z* is the depth from the concrete surface in the y- and *z*-axis directions (unit: mm).

The modified integrated diffusion coefficient for chloride ions under dry and wet cycles is given in Equation (13).

(13) $$D_{f}=\frac{KD_{0}\exp\left[\frac{U}{R}\left(\frac{1}{T_{0}}-\frac{1}{T}\right)\right]}{\left(1+\frac{\alpha }{\omega_{e}})[1+{\left(\frac{1-RH}{1-RH_{c}}\right)}^{4}\right]}$$

The modified integrated diffusion coefficient for chloride ions under static immersion is shown in Equation (14).

(14) $$D_{f}=\frac{D_{0}\exp\left[\frac{U}{R}\left(\frac{1}{T_{0}}-\frac{1}{T}\right)\right]}{\left(1+\frac{\alpha }{\omega_{e}})[1+{\left(\frac{1-RH}{1-RH_{c}}\right)}^{4}\right]}$$

**Figure 8:**
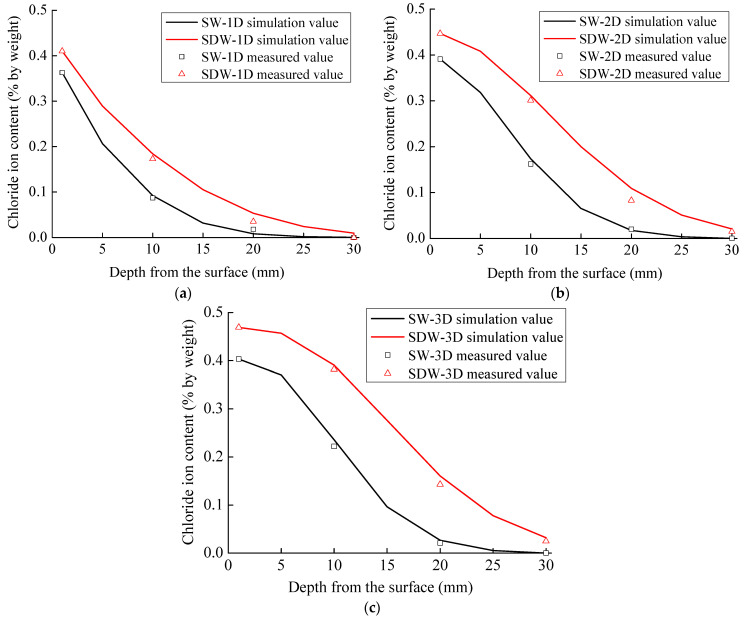
Comparison of chloride diffusion simulation results with experimental results: (**a**) 1D diffusion; (**b**) 2D diffusion; (**c**) 3D diffusion.

We developed a modified multidimensional chloride ion diffusion model using MATLAB R2018b software. The model was applied to simulate concrete specimens exposed to a 90-day immersion, and the comparison between the simulation results in various dimensions and the experimental findings is depicted in Figure 8.

As shown in Figure 8, the predicted chloride ion content in the concrete using the modified multidimensional diffusion model for chloride ions is close to the measured results, and the correlation coefficients are greater than 0.95. The difference between the simulated and measured values decreases progressively as the penetration depth increases. From Figure 8b, it can be seen that for 2D diffusion the simulated values of chloride ion content in concrete specimens under dry and wet cycles have a slight deviation from the measured value at the concrete penetration depth of 20 mm but are basically the same at the depths of 10 mm and 30 mm. This error may be caused by two reasons: Firstly, the large aggregate content in the test powder leads to the small values of some test data. The second reason is that the chloride concentration in the region is low due to the obstruction of coarse aggregate in the chloride ion transport process.

## 4. Conclusions

In order to study the effect of dry and wet cycles in the marine environment on the diffusion of chloride ions in concrete, a multidimensional diffusion experiment of chloride ions in concrete under the dry and wet cycles and static immersion was designed, and the distribution pattern of chloride ions and the changes in the microstructure of the concrete across different time periods were measured. Meanwhile, based on Fick’s second law, a multidimensional diffusion model of chloride ions in concrete under dry and wet cycles and static immersion was established by considering the effects of chloride ion exposure time, environment temperature, relative humidity, and dry and wet cycles, which were simulated by using MATLAB software and then verified using the experiment results; the following conclusions were obtained:

(1) Under the same diffusion conditions, the chloride content of concrete decreases with the increase in penetration depth, and it can be seen that the chloride content of concrete under dry and wet cycling is greater than that of static immersion.
(2) Under the same diffusion conditions, the chloride ion content in concrete increases with the increase in diffusion dimension and exposure time. Chloride ion multidimensional diffusion under dry and wet cycles will exacerbate the chloride ion transport rate in concrete and the penetration depth. The chloride ion content in concrete under 2D and 3D diffusion under dry and wet cycles was 1.09~4.08 times higher than that under 1D diffusion.
(3) The coupled dry and wet cycles and diffusion of chloride ions increase the development of cracks and pores within the concrete structure, which accelerates the rate of chloride ion transport in the concrete.
(4) The correlation coefficients between the simulation results of the multidimensional transport model of chloride ions in concrete under multi-factor coupling and the experimental results were all greater than 0.95, indicating that the model can be used to predict the concentration distribution of chloride ions in concrete.

## Figures and Tables

**Table 1:** Composition and physical properties of cement.

CaO (%)	SiO_2_ (%)	Al_2_O_3_ (%)	Fe_2_O_3_ (%)	SO_3_ (%)	MgO (%)	Cl^−^ (%)	Specific Surface Area (m^2^/kg)	28*d* Compressive Strength (MPa)	Loss on Ignition (%)
65.43	21.05	5.03	3.52	2.31	1.88	0.012	339	58.5	1.34

**Table 2:** Mix proportion of concrete (kg/m^3^).

Cement	Fine Aggregate	Coarse Aggregate	Fly Ash	Slag Powder	Water	Admixture
320	725	1020	60	120	150	4.65

**Table 3:** Chloride ion diffusion conditions.

Group No.	Exposure Environment	Exposure Mode
SW-1D	Static immersion	1D
SW-2D	Static immersion	2D
SW-3D	Static immersion	3D
SDW-1D	Dry and wet cycles	1D
SDW-2D	Dry and wet cycles	2D
SDW-3D	Dry and wet cycles	3D

**Table 4:** Compressive strength and flexural strength of concrete at 28 days.

Property	1	2	3	Average Value
Compress strength/MPa	69.6	68.1	68.9	68.9
Flexural strength/MPa	7.16	6.30	6.12	6.53

## Data Availability

Data will be provided by the corresponding author on request.
